# Effectiveness of the Program to Encourage Active, Rewarding Lives (PEARLS) to reduce depression: a multi-state evaluation

**DOI:** 10.3389/fpubh.2023.1169257

**Published:** 2023-06-09

**Authors:** Matthew Lee Smith, Lesley E. Steinman, Carol N. Montoya, Meghan Thompson, Lixian Zhong, Ashley L. Merianos

**Affiliations:** ^1^Department of Health Behavior, School of Public Health, Texas A&M University, College Station, TX, United States; ^2^Center for Community Health and Aging, Texas A&M University, College Station, TX, United States; ^3^Center for Health Equity and Evaluation Research, Texas A&M University, College Station, TX, United States; ^4^Department of Health Systems and Population Health, Health Promotion Research Center, School of Public Health, University of Washington, Seattle, WA, United States; ^5^Health Foundation of South Florida, Miami, FL, United States; ^6^Sound Generations, Seattle, WA, United States; ^7^School of Pharmacy, Texas A&M University, College Station, TX, United States; ^8^School of Human Services, University of Cincinnati, Cincinnati, OH, United States

**Keywords:** depression, PEARLS, evidence-based program, clinical remission, clinical response, PHQ-9

## Abstract

**Introduction:**

An estimated 15% of community-dwelling older adults have depressive symptoms in the U.S. The Program to Encourage Active, Rewarding Lives (PEARLS) is an evidence-based program for managing late-life depression. PEARLS is a home/community-based collaborative care model delivered by community-based organizations to improve access to quality depression care. Trained staff actively screen for depression to improve recognition, teach problem-solving and activity planning skills for self-management, and connect participants to other supports and services as needed.

**Methods:**

This study examined 2015–2021 data from 1,155 PEARLS participants across four states to assess PEARLS effectiveness to reduce depressive symptoms. The clinical outcomes were measured by the self-reported PHQ-9 instrument to assess changes in depressive symptoms scored as depression-related severity, clinical remission, and clinical response. A generalized estimating equation (GEE) model was fitted to examine changes in composite PHQ-9 scores from baseline to the final session. The model adjusted for participants’ age, gender, race/ethnicity, education level, income level, marital status, number of chronic conditions, and number of PEARLS sessions attended. Cox proportional hazards regression models were conducted to estimate the hazard ratio for improvement of depressive symptoms (i.e., remission or response), while adjusting for the covariates.

**Results:**

PHQ-9 scale scores significantly improved from baseline to their final sessions (mean difference = −5.67, SEM = 0.16, *p* < 0.001). About 35% of participants achieved remission with PHQ-9 score < 5. Compared to participants with mild depression, patients with moderate depression (HR = 0.43, 95%CI = 0.35–0.55), moderately severe depression (HR = 0.28, 95%CI = 0.21–0.38), and severe depression (HR = 0.22 95%CI = 0.14–0.34) were less likely to experience clinical remission with PHQ-9 score < 5, while adjusting for the covariates. About 73% achieved remission based on no longer having one or both cardinal symptoms. Compared to participants with mild depression, patients with moderate depression (HR = 0.66, 95%CI = 0.56–0.78), moderately severe depression (HR = 0.46, 95%CI = 0.38–0.56), and severe depression (HR = 0.38, 95%CI = 0.29–0.51) were less likely to experience clinical remission, while adjusting for the covariates. Nearly 49% of participants had a clinical response or a ≥ 50% decrease in PHQ-9 scores over time. There were no differences between the severity of depression groups based on the time to clinical response.

**Discussion:**

Findings confirm that PEARLS is an effective program to improve depressive symptoms among older adults in diverse real-world community settings and can be a more accessible option for depressive older adults who are traditionally underserved by clinical care.

## 1. Introduction

Depression is a prevalent mental health disorder impacting older adults in the United States (U.S.), with trends remaining relatively stable over the past two decades (1). According to the Centers for Disease Control and Prevention in 2019, approximately 19% of the older adult population had symptoms of depression, with about 12, 4, and 3% of adults ages 65 years and older having mild, moderate, and severe symptoms (2). Among older adults ages 75 years and older, the pooled prevalence of major depression is about 7% and depressive disorders is about 17% (3). Older adults of lower income, with multiple chronic conditions, and/or functional limitations for activities of daily living are risk factors for clinical depression (4–6). Concerning chronic conditions, depressive disorders among older adults have been associated with increased prevalence of asthma, arthritis, cardiovascular disease, cancer, diabetes, and stroke (7).

Clinically significant depression is currently underdiagnosed and thus undertreated among older adults (8, 9). This is concerning especially since depression is an independent predictor of mortality among older adults, with late-life depression increasing mortality risk by 34% (10). Risk factors and symptoms of depression may be overlooked, misdiagnosed as dementia, or mistakenly believed to be a natural response to life changes or disease diagnoses (11–13). Prior research indicates that about 8-in-10 of adults have an undiagnosed depression status, with an economic burden of about $10,000 lost quality-adjusted life years per adult with undiagnosed depression compared to those without undiagnosed depression (14). When compared to younger- and middle-aged adults, older adults are less likely to seek mental health care, and when they receive care, they are less likely to seek specialty care for mental health disorders (15).

Depression treatment for older adults typically focuses on pharmacological treatment (16). While effective, anti-depressant treatments do come with some risks, such as increased fall risk (17) and challenges prescribing medications due to physiological changes that occur during the aging process (18). Fortunately, effective non-pharmacological treatments exist to treat depression among older adults (19) and overcome barriers to healthcare access including inadequate outreach and professional training (20). Specifically, recommendations for treating depression among older adults include home- and community-based depression care management interventions with an individualized cognitive behavioral therapy component (21). One evidence-based program for late-life depression, the Program to Encourage Active, Rewarding Lives (PEARLS), was developed and researched by the Health Promotion Research Center at the University of Washington in partnership with local community-based social service organizations (22).

PEARLS is a home/community-based collaborative care model delivered by community-based organizations to reduce depression among older adults who are isolated, functionally impaired, live with multiple chronic conditions, and/or are of lower socioeconomic status (e.g., social service recipients) (23). Previous research has demonstrated the effectiveness of PEARLS to reduce clinically significant depressive symptoms via randomized controlled trials with older adults with minor depression or dysthymia as well as adults with epilepsy and co-cooccurring major depression (24–26). Beyond this efficacy research, implementation research has been conducted to evaluate the public health impact of PEARLS via the RE-AIM Framework (27), studies have identified strategies and adaptations to make PEARLS more effective for and accessible to older adults of color, including linguistically diverse populations (28, 29). Despite the previously documented effectiveness of PEARLS, fewer studies have examined PEARLS participant attributes and program effectiveness in real-world implementations across multiple states or its ability to reduce depression using different clinical criteria including clinical remission and response. Therefore, the purposes of this four-state investigation were to: (1) identify the baseline characteristics of participants entering PEARLS overall and stratified by their depressive symptoms; (2) assess the effectiveness of PEARLS to reduce depressive symptoms from baseline to the final session; and (3) evaluate the rate of achieving clinical remission and clinical response from baseline to the final session.

## 2. Methods

### 2.1. Intervention

PEARLS is a home/community-based depression care management program like Healthy IDEAS (30) and Beat the Blues (31) in which existing community-based organization staff are trained to provide psychoeducation, support, and brief behavioral interventions to improve access to and quality of depression care for older adults facing barriers to clinical care. PEARLS includes active screening for depression to improve poor recognition of late-life depression, measurement-based outcomes (regular depression symptom monitoring), and clinical supervision to strengthen team-based care that adjusts as needed to best support older adults over the course of care. PEARLS is delivered based on evidence-based guidelines, using a program manual and forms. The program structure includes an initial assessment, six to eight in-person sessions, a final assessment, and three follow-up phone calls. During a six-month period, consisting of the one-on-one sessions and phone support, trained PEARLS coaches (or “counselors”) empower individuals to improve their depressive symptoms and self-efficacy with a three-pillar approach: (1) learning problem solving techniques; (2) becoming more socially and physically active; and (3) engaging in more pleasant activities. This three-pillar approach is proven to reduce depressive symptoms (23, 26). The program is typically delivered in the client’s home, which supports engagement and retention of underserved older adults who may be depressed but less able to seek treatment due to physical impairments and medical comorbidities (23). PEARLS coaches undergo formal training (32) and contact the clients with in-home visits as well as by phone. This ensures that participants feel comfortable expressing any problems and barriers that they are experiencing, which builds rapport with the counselors delivering the program. The PEARLS program trains front-line providers (e.g., community health workers, social workers) who are employed by social service organizations that act as “safety nets” for underserved communities who have lower healthcare access. PEARLS coaches are not required to have a mental health background or other formal credentials; rather, the training, structured delivery guidelines, clinical supervision by psychiatrists or other clinicians provide ongoing support and consultation for PEARLS participants with complex healthcare needs. Clinical supervision has shown consistent fidelity of PEARLS implementation (33).

### 2.2. Participants and procedures

This collaborative effort examined data from PEARLS participants across four Administration for Community Living (ACL)-funded states (i.e., Florida, Maryland, Texas, and Washington) to assess the effectiveness of PEARLS to reduce depressive symptoms. PEARLS participants are mostly older adults ages ≥50 years who are of low-income and have clinical depressive symptoms determined by using the reliable and valid Patient Health Questionnaire-9 (PHQ-9) (34). PEARLS routine practice requires the PHQ-9 being administered at each session for monitoring, tracking and psychoeducation. PEARLS participants frequently meet the Diagnostic and Statistical Manual of Mental Disorders (DSM-5) criteria for minor depression, major depression, and persistent depressive disorder (35). Ineligibility criteria for participation in PEARLS includes people with moderate to severe cognitive impairment and those who are functionally impaired by serious mental health illnesses or substance use disorders.

The date for participant enrollment ranged from 05/18/2015 to 02/17/2021. Of the 1,856 potential PEARLS participants, 360 (19.4%) were deemed ineligible after initial screening and 60 (3.2%) were not screened for eligibility. Of the 1,436 eligible PEARLS participants, 1,373 (95.6%) completed the 9-item Patient Health Questionnaire (PHQ-9) during their baseline session. Of the 1,373 participants with an initial PHQ-9 score, 1,225 (89.2%) subsequently completed at least one PHQ-9 screening. Prior to analyses, we excluded an additional 2.3% (*n* = 28) of participants ages <50 years. Because PEARLS is intended for those with depression, an additional 3.5% (*n* = 42) of participants with initial PHQ-9 scores of 0–4 (i.e., indicative of no/minimal depressive symptoms) were omitted. Therefore, the primary analytic sample for this investigation included the 1,155 PEARLS participants ages ≥50 years with a baseline score indicative of depression and at least two PHQ-9 scores. The Texas A&M University Institutional Review Board classified this secondary data analysis as not human subjects research (IRB#: 2020–1244).

### 2.3. Measures

#### 2.3.1. PHQ-9

The PHQ-9 is a self-reported tool that assesses depressive symptoms over the past 2 weeks (34). The nine questions align with the DSM-5 diagnostic criteria to identify the severity of depression (35), and cover emotional (e.g., mood), cognitive (e.g., poor concentration), and somatic (e.g., trouble sleeping) depressive symptoms. Each of the nine items is scored on a 4-point Likert scale from 0 (“not at all”), 1 (“several days”), 2 (“more than half the days)” to 3 (“nearly every day”) (34). For this study, we examined the PHQ-9 in different ways to assess changes in depression-related severity, clinical remission, and clinical response.

The PHQ-9 can be assessed continuously where the items are summed to create a scale score from 0 to 27, with higher scores indicating more depressive symptoms (34). In the current sample, this 9-item scale had a Cronbach’s alpha of 0.628. When used as a severity measure, the PHQ-9 scale scores can be assessed categorically by severity of depression thresholds: (1) 0–4 (i.e., no/minimal); (2) 5–9 (i.e., mild); (3) 10–14 (i.e., moderate); (4) 15–19 (i.e., moderately severe); and (5) 20–27 (i.e., severe).

PHQ-9 clinical outcomes can be interpreted as: (1) clinical remission, which is indicative of not needing depression treatment and defined as either (a) having a PHQ-9 scale score < 5 or (b) no longer having one or both cardinal symptoms of “little interest” or “depressed mood” more than half the time or about every day, irrespective of their composite PHQ-9 score; and (2) clinical response, which was defined as a clinically significant improvement with ≥50% decline in PHQ-9 scale scores from pre-treatment/baseline to the final PEARLS session (34, 36). Additionally, at least one of the cardinal depressive symptoms [i.e., the first two PHQ-9 symptoms of “little interest or pleasure in doing things” (anhedonia) and “feeling down, depressed, or hopeless” (depressed mood)] must be endorsed as “more than half the days” or “nearly every day” (34) to meet the DSM criteria for depression (35).

#### 2.3.2. Covariates

The following sociodemographic characteristics of participants were assessed: age; gender; race and ethnicity (non-Hispanic White, non-Hispanic Black, non-Hispanic Other/Multiracial, and Hispanic); education level (less than high school/GED, high school graduate/GED, some college or technical school, and college graduate or higher); income level (<$15,000, $15,000–$24,999, ≥$25,000); marital status (married or partnered, widowed, divorced or separated, and single or never married); and number of chronic health conditions (e.g., arthritis, cancer, heart disease, obesity). The non-Hispanic Other category included Asian, American Indian or Alaska Native, Native Hawaiian or Pacific Islander, and Multiracial. Concerning PEARLS participation, participant characteristics also included number of PEARLS sessions attended (range 1–8) and successful completion of the program, which was defined as attending 6–8 sessions.

### 2.4. Statistical analysis

Descriptive statistics were performed to identify sample characteristics. Independent t-tests and one-way analyses of variance (ANOVAs) were performed to assess differences between categorical participant characteristics based on baseline PHQ-9 scores. It should be noted that there were missing data for gender, education level, income level, and marital status. Rather than omit cases, missing values for these covariates were included in sample frequencies. Differences between groups were assessed, and no differences were identified. Pearson correlations were performed to assess the strength of associations between the continuous participant characteristics based on baseline PHQ-9 scores. Then, an adjusted generalized estimating equation (GEE) model was fitted with an identity link function and exchangeable correlation structure for repeated-measures linear regression to examine composite PHQ-9 scores continuously from baseline to the final PEARLS session. Covariates were included in the model to test the overall effects of PEARLS by adjusting for participants’ age, gender, race/ethnicity, education level, income level, marital status, number of chronic conditions, and number of PEARLS sessions attended. The mean difference, standard error of the mean (SEM), and *p-*values are presented for the adjusted GEE model.

To assess the three time-to-event outcomes of clinical remission and response, we employed the Kaplan–Meier method to illustrate the cumulative probability of time to clinical remission and time to having a clinical response over the eight PEARLS sessions based on participants’ severity of depression at baseline. Participants were censored in each Kaplan–Meier survival curve if they did not achieve either remission or response during PEARLS. Then, we performed three Cox proportional hazards regression models for each primary outcome to estimate the hazard ratio for improvement of depressive symptoms (i.e., remission or response), while adjusting for the covariates. Participants with mild depression at baseline were used as the reference group, and adjusted hazard ratios (HRs), 95% confidence intervals (CIs), and *p*-values are presented for the three adjusted Cox models. Data were analyzed as intent-to-treat using SPSS (version 28.0).

## 3. Results

On average, PEARLS participants were age 71.8 (±9.8) years, ranging from 50 to 101 years (Table 1). Approximately 76% were female, and the majority were non-Hispanic White (60.2%) followed by non-Hispanic Black (20.7%), non-Hispanic Other/Multiracial (14.2%) and Hispanic (4.9%). About 15% of participants had a high school education/GED or less, whereas 16.5% had a college education or more. The majority (38.5%) of participants had annual household incomes <$15,000. Concerning marital status, 26.1 and 24.6% were divorced or separated and widowed, respectively. On average, PEARLS participants self-reported 2.8 (±2.4) chronic health conditions. In addition to self-reported depression, the most frequently reported conditions included hypertension (40.5%), arthritis/rheumatic disease (28.5%), chronic pain (26.7%), high cholesterol (26.5%), heart disease (24.8%), and asthma/emphysema/other breathing problems (17.1%).

**Table 1:** PEARLS participant characteristics overall and based on baseline PHQ-9 scale scores.

Participant characteristic	Overall (*N* = 1,155) *n* (%)^a^	Baseline PHQ-9 score, *M* (SD)	*p*-value
Age, *M* (SD)^b^	71.8 (9.8)	−0.131^b^	<0.001
Gender			0.288
Female	881 (76.3)	13.19 (4.60)	
Male	250 (21.6)	12.72 (4.55)	
Unknown	24 (2.1)	13.71 (4.38)	
Race/Ethnicity			<0.001
Non-Hispanic White	695 (60.2)	13.21 (4.67)	
Non-Hispanic Black	239 (20.7)	12.18 (3.99)	
Non-Hispanic Other/Multiracial	164 (14.2)	13.99 (5.13)	
Hispanic	57 (4.9)	12.93 (3.63)	
Education level			0.754
Less than high school/GED	169 (14.6)	12.85 (4.08)	
High school graduate/GED	303 (26.2)	12.96 (4.40)	
Some college or technical	336 (29.1)	13.32 (4.98)	
College graduate or higher	190 (16.5)	12.98 (4.63)	
Unknown	157 (13.6)	13.29 (4.56)	
Income level			0.037
<$15,000	445 (38.5)	13.35 (4.54)	
$15,000–$24,999	233 (20.2)	12.65 (4.54)	
≥$25,000	161 (13.9)	12.42 (4.89)	
Unknown	316 (27.4)	13.41 (4.49)	
Marital status			0.053
Married or partnered	232 (20.1)	12.69 (4.49)	
Widowed	284 (24.6)	12.61 (4.63)	
Divorced or separated	301 (26.1)	13.52 (4.80)	
Single, never married	175 (15.1)	13.30 (4.45)	
Unknown	163 (14.1)	13.51 (4.30)	
No. chronic conditions, *M* (SD)	2.8 (2.4)	−0.013^b^	0.671
Severity of depression based on PHQ-9 score			<0.001
Mild (5–9 score)	279 (24.1)	7.47 (1.29)	
Moderate (10–14 score)	463 (40.1)	11.98 (1.39)	
Moderately Severe (15–19 score)	292 (25.3)	16.70 (1.40)	
Severe (20–27 score)	121 (10.5)	21.64 (1.71)	
No. PEARLS sessions attended, *M* (SD)	5.8 (2.4)	−0.090^b^	0.002
PEARLS successful completion			0.006
No (0–5 sessions)	416 (36.0)	13.60 (4.71)	
Yes (6–8 sessions)	739 (64.0)	12.81 (4.50)	

PHQ-9, Patient Health Questionnaire-9; GED, general education development; PEARLS, Program to Encourage Active, Rewarding Lives.

^a^n (%) unless noted otherwise.

^b^Pearson correlation coefficient.

On average, participants attended 5.8 (±2.4) PEARLS sessions. Nearly two-thirds (64.0%) of participants successfully completed PEARLS and attended 6–8 sessions. PEARLS participants represented four states, with 67.4% living in Florida (*n* = 779), followed by 19.3% in Maryland (*n* = 223), 8.3% in Texas (*n* = 96), and 4.9% in Washington (*n* = 57). Participants were primarily referred to the program by Area Agencies on Aging (42.0%), followed by a nurse or social worker (9.0%), self-referral (8.7%), physician or health care professional (7.1%), or outreach activity (6.6%).

### 3.1. Sample characteristics based on baseline PHQ-9 scale scores

The average baseline PHQ-9 score for participants was 13.10 (±4.59), with scores ranging from 5 to 27. Concerning severity of depression based on baseline PHQ-9 scale scores, 24.1% of participants had mild depression, 40.1% had moderate depression, 25.3% had moderately severe depression, and 10.5% had severe depression (see Table 1).

Participants’ age (*r* = −0.131, *p* < 0.001) and number of PEARLS sessions attended (*r* = −0.090, *p* = 0.002) negatively correlated with baseline PHQ-9 scale scores (see Table 1). One-way ANOVA test results indicated that baseline PHQ-9 scale scores differed by race/ethnicity (*p* < 0.001), income level (*p* = 0.037), and severity of depression (*p* < 0.001). Independent t-test results indicated that participants who had successful PEARLS completion defined as attending 6–8 sessions had lower mean baseline PHQ-9 scores compared to participants who attended 0–5 sessions (*p* = 0.006).

### 3.2. Changes in PHQ-9 scale scores from baseline to final PEARLS session

The average PHQ-9 scale score at participants’ final sessions was 7.42 (±5.33), with scores ranging from 0 to 25. Table 2 reports the adjusted GEE model results for changes in PHQ-9 scale scores from baseline to final session among PEARLS participants. Specifically, PHQ-9 scale scores significantly improved from baseline to their final sessions (mean difference = −5.67, SEM = 0.16, *p* < 0.001). Participants with improved PHQ-9 scores from baseline to their final sessions were older (*β* = −0.05, *p* < 0.001) and attended a higher mean number of PEARLS sessions (*β* = −0.52, *p* < 0.001). Non-Hispanic Black participants (mean difference = −1.44, *p* < 0.001) and participants with an income ≥$25,000 (mean difference = −1.02, *p* = 0.010) had significantly improved PHQ-9 scale scores compared to participants who were non-Hispanic White and with an income <$15,000, respectively. Conversely, participants who were divorced or separated (mean difference = 0.75, *p* = 0.031) and single or never married (mean difference = 0.81, *p* = 0.040) had poorer PHQ-9 scale score improvement compared to participants who were married or partnered.

**Table 2:** Changes in PHQ-9 scale scores from baseline session to final session among PEARLS participants.

	Baseline PHQ-9 score, *M* (SD)	Final PHQ-9 score, *M* (SD)	Adjusted GEE model Mean difference (SEM)^a^	*p*-value
Overall PHQ-9 depression score (range 0–27)	13.10 (4.59)	7.42 (5.33)	−5.67 (0.16)	<0.001
Age	–	–	−0.05	<0.001
Gender				
Female	13.19 (4.60)	7.37 (5.39)	Ref	Ref
Male	12.72 (4.55)	7.41 (5.11)	−0.33	0.249
Unknown	13.71 (4.38)	9.29 (5.21)	0.44	0.561
Race/Ethnicity				
Non-Hispanic White	13.21 (4.67)	7.75 (5.33)	Ref	Ref
Non-Hispanic Black	12.18 (3.99)	6.37 (4.88)	−1.44	<0.001
Non-Hispanic Other/Multiracial	13.99 (5.13)	7.74 (5.98)	0.08	0.845
Hispanic	12.93 (3.63)	6.84 (4.46)	−0.59	0.190
Education level				
Less than high school/GED	12.85 (4.08)	7.58 (4.70)	Ref	Ref
High school graduate/GED	12.96 (4.40)	6.87 (5.26)	−0.55	0.106
Some college or technical	13.32 (4.98)	7.46 (5.42)	−0.03	0.931
College graduate or higher	12.98 (4.63)	6.97 (5.06)	−0.40	0.317
Unknown	13.29 (4.56)	8.78 (5.98)	0.02	0.965
Income level				
<$15,000	13.35 (4.54)	7.97 (5.30)	Ref	Ref
$15,000–$24,999	12.65 (4.54)	6.86 (5.09)	−0.58	0.072
≥$25,000	12.42 (4.89)	6.54 (5.14)	−1.02	0.010
Unknown	13.41 (4.49)	7.52 (5.54)	−0.26	0.409
Marital status				
Married or partnered	12.69 (4.49)	6.83 (4.55)	Ref	Ref
Widowed	12.61 (4.63)	6.98 (5.35)	0.34	0.331
Divorced or separated	13.52 (4.80)	7.63 (5.54)	0.75	0.031
Single, never married	13.30 (4.45)	8.23 (5.43)	0.81	0.040
Unknown	13.51 (4.30)	7.78 (5.69)	0.56	0.183
No. chronic conditions	–	–	0.01	0.888
No. PEARLS sessions attended	–	–	−0.52	<0.001

PHQ-9, Patient Health Questionnaire-9; GED, general education development; PEARLS, Program to Encourage Active, Rewarding Lives; Ref, reference category.

^a^GEE model adjusting for participant age, gender, race/ethnicity, education level, income level, marital status, number of chronic conditions, and number of PEARLS sessions attended. Bold font indicates statistical significance *p* < 0.05.

### 3.3. Time to clinical remission from baseline to final session

**Figure 1:**
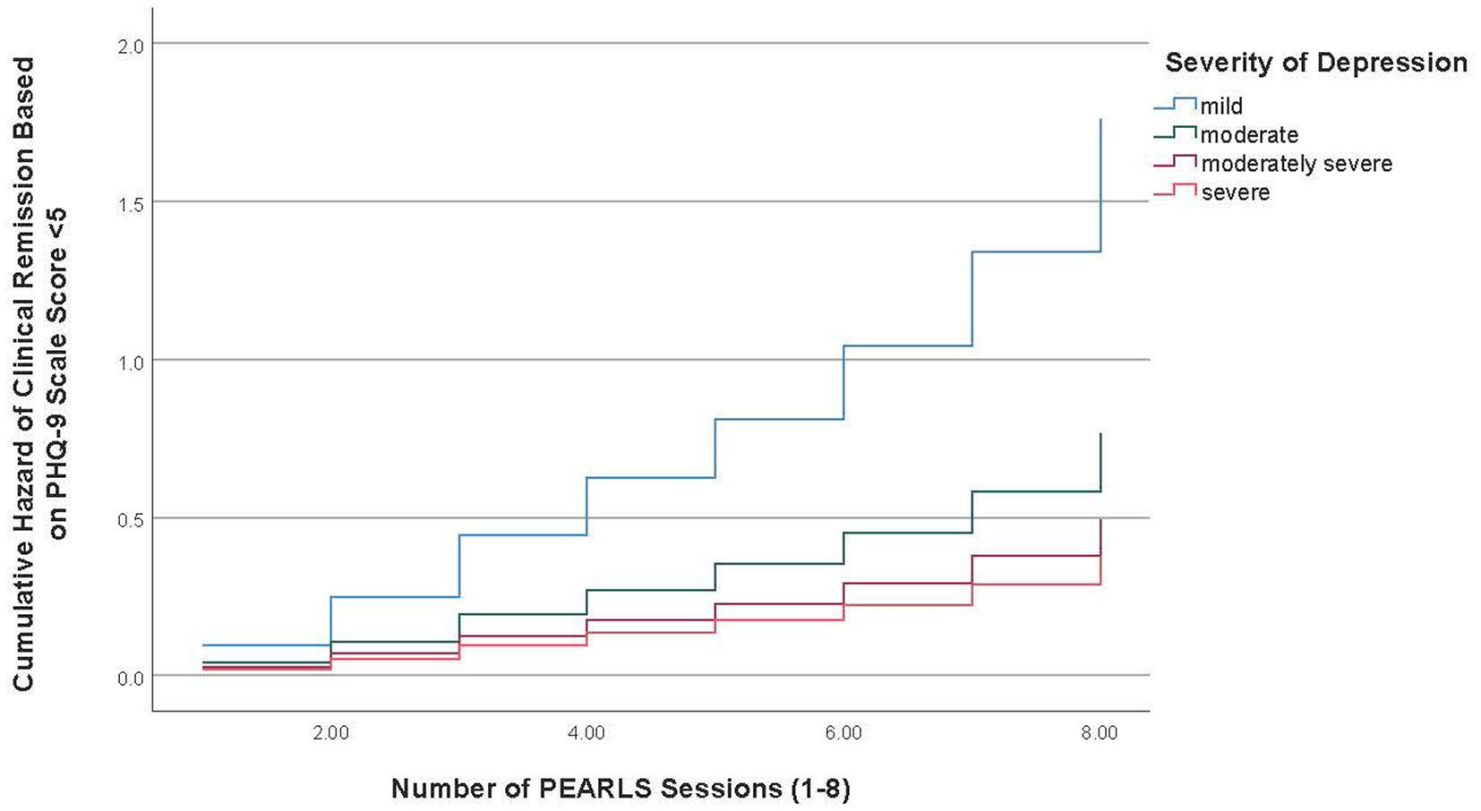
Time to clinical remission curve for severity of depression groups (PHQ-9 score < 5).

**Figure 2:**
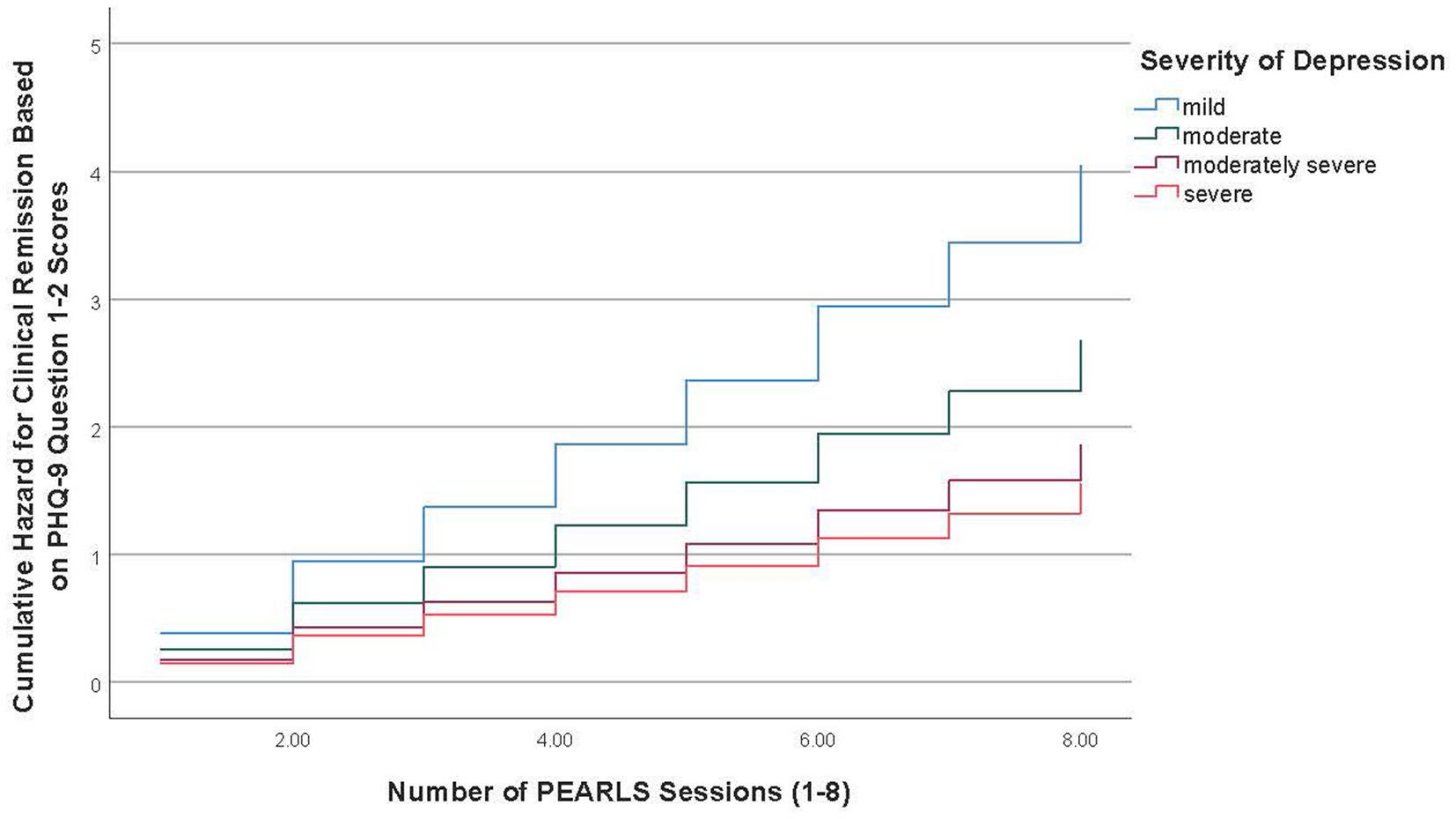
Time to clinical remission curve for severity of depression groups (cardinal symptoms).

Overall, from baseline to final session, 34.9% of participants (*n* = 403) achieved remission based on PHQ-9 scale score < 5 criteria and 73.3% (*n* = 847) achieved remission based on the criteria of no longer having one or both symptoms included in items 1–2 (i.e., little interest or depressed mood) for more than half the time or about every day. Table 3 reports the adjusted Cox regression model results for the time to clinical remission, based on the two criteria, from baseline to the final session. The time to remission curves for mild depression versus the three other severity of depression groups is depicted graphically in Figures 1, 2.

**Table 3:** Time to clinical remission and clinical response from baseline to final session based on baseline depressive severity.

	Clinical remission based on PHQ-9 scale score < 5		Clinical remission based on PHQ-9 Q1-Q2 scores		Clinical response based on ≥ 50% decrease in PHQ-9 scale score	
	HR (95%CI)^a^	*p*-value	HR (95%CI)^a^	*p*-value	HR (95%CI)^a^	*p*-value
Baseline severity of depression						
Mild (PHQ-9 5–9 score)	Ref	Ref	Ref	Ref	Ref	Ref
Moderate (PHQ-9 10–14 score)	0.43 (0.35–0.55)	<0.001	0.66 (0.56–0.78)	<0.001	0.98 (0.79–1.21)	0.840
Moderately Severe (PHQ-9 15–19 score)	0.28 (0.21–0.38)	<0.001	0.46 (0.38–0.56)	<0.001	1.07 (0.85–1.37)	0.559
Severe (PHQ-9 20–27 score)	0.22 (0.14–0.34)	<0.001	0.38 (0.29–0.51)	<0.001	1.11 (0.81–1.52)	0.537
Age	1.00 (0.99–1.01)	0.519	1.00 (0.99–1.01)	0.743	1.00 (0.99–1.01)	0.561
Gender						
Female	Ref	Ref	Ref	Ref	Ref	Ref
Male	0.90 (0.70–1.16)	0.411	0.85 (0.72–1.01)	0.066	0.85 (0.68–1.05)	0.127
Unknown	0.41 (0.17–1.01)	0.051	0.68 (0.40–1.14)	0.142	0.48 (0.22–1.03)	0.060
Race/Ethnicity						
Non-Hispanic White	Ref	Ref	Ref	Ref	Ref	Ref
Non-Hispanic Black	1.21 (0.94–1.56)	0.147	1.07 (0.89–1.27)	0.489	1.31 (1.05–1.63)	0.017
Non-Hispanic Other/Multiracial	1.32 (0.97–1.78)	0.078	1.15 (0.93–1.44)	0.204	1.27 (0.97–1.65)	0.079
Hispanic	0.86 (0.52–1.43)	0.565	0.79 (0.57–1.11)	0.182	1.04 (0.71–1.51)	0.847
Education level						
Less than high school/GED	Ref	Ref	Ref	Ref	Ref	Ref
High school graduate/GED	1.95 (1.37–2.78)	<0.001	1.14 (0.91–1.43)	0.267	1.52 (1.14–2.01)	0.004
Some college or technical	1.51 (1.05–2.16)	0.025	1.36 (1.08–1.70)	0.008	1.26 (0.94–1.69)	0.116
College graduate or higher	1.68 (1.13–2.49)	0.010	1.38 (1.07–1.78)	0.013	1.48 (1.07–2.04)	0.018
Unknown	1.20 (0.76–1.88)	0.441	1.24 (0.92–1.66)	0.153	1.03 (0.70–1.50)	0.888
Income level						
<$15,000	Ref	Ref	Ref	Ref	Ref	Ref
$15,000–$24,999	1.31 (0.99–1.72)	0.054	1.02 (0.85–1.23)	0.828	1.22 (0.97–1.54)	0.096
≥$25,000	1.70 (1.25–2.33)	<0.001	1.11 (0.89–1.39)	0.365	1.47 (1.11–1.93)	0.006
Unknown	1.33 (1.01–1.76)	0.042	1.10 (0.91–1.34)	0.316	1.46 (1.16–1.85)	0.001
Marital status						
Married or partnered	Ref	Ref	Ref	Ref	Ref	Ref
Widowed	1.17 (0.87–1.58)	0.304	0.94 (0.76–1.17)	0.597	0.94 (0.73–1.22)	0.661
Divorced or separated	0.99 (0.74–1.33)	0.948	1.06 (0.86–1.30)	0.598	0.94 (0.73–1.20)	0.597
Single, never married	0.75 (0.51–1.09)	0.133	0.93 (0.73–1.18)	0.539	0.78 (0.58–1.06)	0.117
Unknown	1.17 (0.81–1.69)	0.394	1.05 (0.81–1.35)	0.732	0.92 (0.68–1.25)	0.610
No. chronic conditions	0.98 (0.94–1.03)	0.469	0.99 (0.96–1.03)	0.694	0.99 (0.95–1.03)	0.521

PHQ-9, Patient Health Questionnaire-9; GED, general education development; PEARLS, Program to Encourage Active, Rewarding Lives; HR, hazard ratio; CI, confidence interval; Ref, reference category; No, number. ^a^Cox proportional hazard models adjusting for participant age, gender, race/ethnicity, education level, income level, marital status, and number of chronic conditions. Bold font indicates statistical significance *p* < 0.05.

The mean duration for participants with mild depression to achieve remission based on PHQ-9 scale score < 5 criteria was 4.67 sessions (95%CI = 4.33–5.02) followed by 6.36 sessions (95%CI = 6.14–6.59) for participants with moderate depression, 6.96 sessions (95%CI = 6.72–7.20) for participants with moderately severe depression, and 7.12 sessions (95%CI = 6.75–7.49) for participants with severe depression. Cox regression model results showed the time to clinical remission based on the PHQ scale score < 5 criteria was significantly different among all severity of depression groups. Specifically, compared to participants with mild depression, patients with moderate depression (HR = 0.43, 95%CI = 0.35–0.55), moderately severe depression (HR = 0.28, 95%CI = 0.21–0.38), and severe depression (HR = 0.22 95%CI = 0.14–0.34) were less likely to experience clinical remission, while adjusting for the covariates (see Table 3). Compared to participants in the lowest education level, participants with higher education levels of high school graduate/GED (HR = 1.95, 95%CI = 1.37–2.78), some college or technical school (HR = 1.51, 95%CI = 1.05–2.16), and college degree or higher (HR = 1.68, 95%CI = 1.13–2.49) were more likely to achieve clinical remission. Compared to participants in the lowest income level, participants with a higher income level of ≥$25,000 (HR = 1.70, 95%CI = 1.25–2.33) were more likely to experience clinical remission.

The mean duration for participants with mild depression to achieve remission based on the criteria of no longer having one or both symptoms included in items 1–2 (i.e., little interest or depressed mood) for more than half the time or about every day was 2.54 sessions (95%CI = 2.32–2.75), followed by 3.53 sessions (95%CI = 3.30–3.76) for participants with moderate depression, 4.44 sessions (95%CI = 4.12–4.75) for participants with moderately severe depression, and 4.65 sessions (95%CI = 4.14–5.16) for participants with severe depression. Cox regression model results showed the time to clinical remission based on the criteria of no longer having one or both symptoms for PHQ-9 items 1–2 was significantly different among all severity of depression groups. Specifically, compared to participants with mild depression, patients with moderate depression (HR = 0.66, 95%CI = 0.56–0.78), moderately severe depression (HR = 0.46, 95%CI = 0.38–0.56), and severe depression (HR = 0.38, 95%CI = 0.29–0.51) were less likely to experience clinical remission, while adjusting for the covariates (see Table 3). Compared to participants in the lowest education level, participants with higher education levels of some college or technical school (HR = 1.36, 95%CI = 1.08–1.70) and college degree or higher (HR = 1.38, 95%CI = 1.07–1.78) were more likely to experience clinical remission.

### 3.4. Resource time to clinical response from baseline to final session based on baseline severity of depression

**Figure 3:**
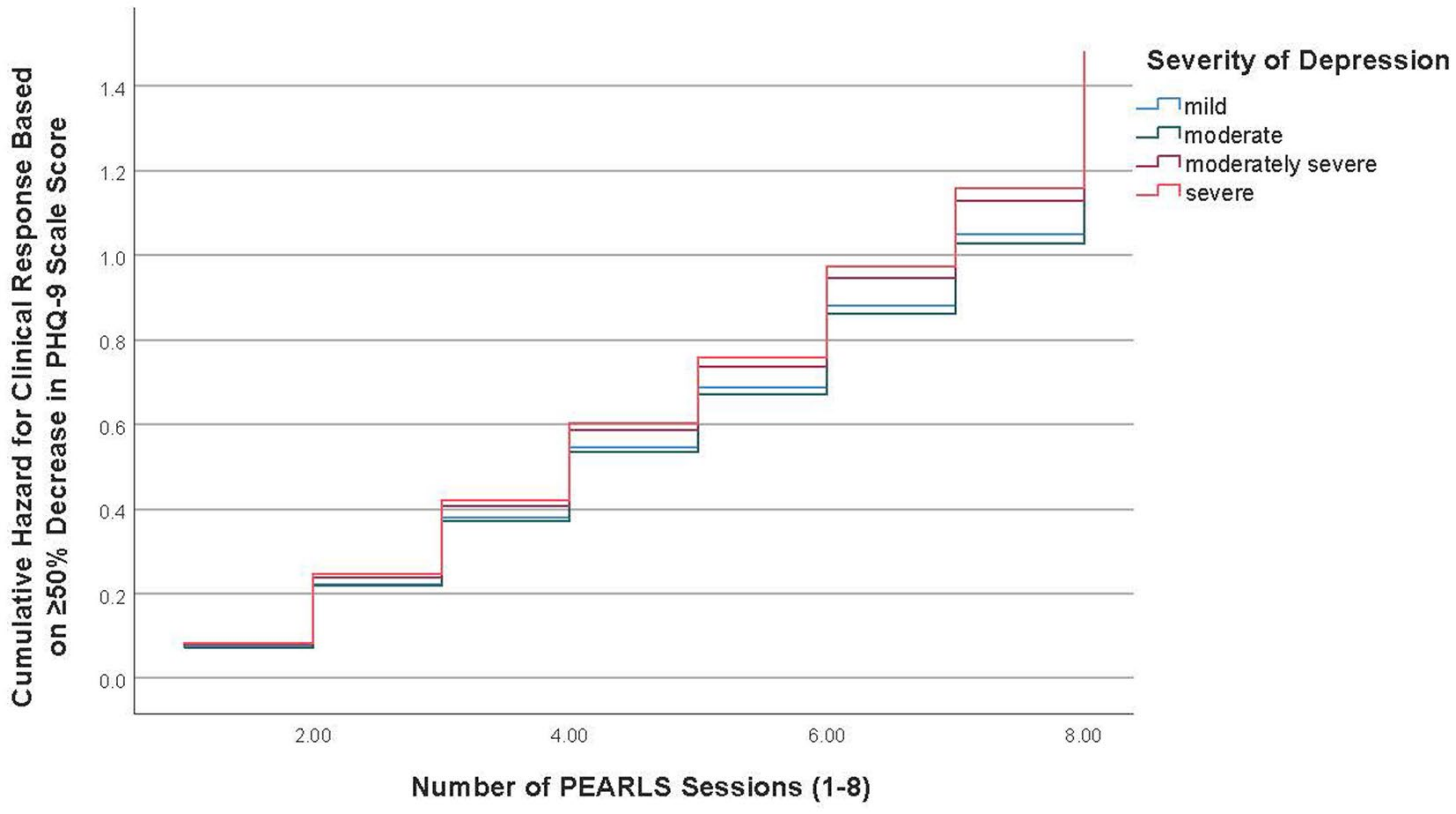
Time to clinical response curve for severity of depression groups (PHQ-9 ≥ 50% decrease).

Overall, from baseline to final session, 48.7% of participants (*n* = 562) had a clinical response or a ≥ 50% decrease in PHQ-9 scale scores over time. Table 3 also reports the adjusted Cox regression model results for the time to clinical response based on ≥50% decrease in PHQ-9 scale scores. Figure 3 illustrates the time to clinical response curve for mild depression versus the three other severity of depression groups.

The mean duration for participants with mild depression to have a clinical response based on the criteria of ≥50% decrease in PHQ-9 scale scores was 5.16 sessions (95%CI = 4.83–5.49), followed by 5.29 sessions (95%CI = 5.02–5.55) for participants with moderate depression, 5.21 sessions (95%CI = 4.89–5.52) for participants with moderately severe depression, and 5.02 sessions (95%CI = 4.48–5.56) for participants with severe depression. Cox regression model results showed no differences between the severity of depression groups based on the time to clinical response based on the criteria of ≥50% decrease in PHQ-9 scale scores (see Table 3). Compared to non-Hispanic White participants, non-Hispanic Black participants (HR = 1.31, 95%CI = 1.05–1.63) were more likely to achieve a clinical response. Compared to participants in the lowest education level, participants with higher education levels of high school graduate/GED (HR = 1.52, 95%CI = 1.14–2.01) and college degree or higher (HR = 1.48, 95%CI = 1.07–2.04) were more likely to achieve a clinical response. Compared to participants in the lowest income level, participants with a higher income level of ≥$25,000 (HR = 1.47, 95%CI = 1.11–1.93) were more likely to achieve a clinical response.

## 4. Discussion

This study examined the effectiveness of PEARLS implemented across four U.S. states. Findings reinforce the robustness of PEARLS to reach diverse participants and effectively improve depressive symptoms among older adults in “real world” community settings outside of research studies (24, 28). This study was unique in that it used multiple PHQ-9 scoring definitions and thresholds to assess PEARLS’s effectiveness to reduce depressive symptoms among older adults. On average, participants reported more than a 5-point decrease in PHQ-9 scores from baseline to final session, with large proportions of participants achieving clinical remission and clinical response.

In the current sample, 25.3 and 10.5% of participants reported moderately severe and severe depression at baseline, respectively, and significant differences were observed in baseline depression levels based on participants’ age, race/ethnicity, and income levels. These findings align with prior research that older adults with higher odds of major depression were ages 55–69 years, previously married, and had an income <$20,000, but Black or African American participants were associated with lower odds of major depression (37). The current study also shows that socioeconomic status (i.e., education and income levels) was associated with increased odds of clinical remission and clinical response among PEARLS participants, which suggests the value of PEARLS for individuals without accessible or affordable mental health services. Offering community-based depression care can reduce mental health inequities by improving service availability and utilization among older adults typically underserved by clinical care. Recent COVID-19 adaptations to PEARLS complements its existing in-person national delivery infrastructure by improving adoption though distance training and telePEARLS (i.e., delivered by telephone or video-conferencing sessions) (22, 29, 38).

The current study builds upon growing evidence utilizing survival analysis to assess depressive symptoms, using the PHQ-9 from baseline to after treatment (24, 28, 39, 40). In addition to examining the PHQ-9 as a continuous variable, this study also used survival analysis to assess participants’ clinical response to the intervention (i.e., ≥50% decline in PHQ-9 scores from baseline) and achievement of clinical remission using two definitions (i.e., reduced PHQ-9 scores to <5 and no longer having one of the cardinal symptoms included in items 1–2). Significant clinical improvements were observed across models using different PHQ-9 scoring criteria. More specifically, 73.3% of participants achieved clinical remission in terms of no longer having one or both cardinal depressive symptoms, 48.7% achieved clinical response, and 34.9% achieved clinical remission in terms of a PHQ-9 score < 5 from baseline. These differences by clinical definitions highlight the importance of PEARLS delivery sites selecting the most appropriate and applicable scoring criteria to identify program success. Sites are encouraged to determine the relative importance of participants decreasing their depressive symptoms (i.e., clinical response) versus achieving minimal symptoms (i.e., clinical remission) as a result of the intervention (41). Selected definitions for clinically meaningful improvements should align with organizational priorities and criteria for billing and reimbursement models.

The ability of PEARLS to achieve clinical improvements via reductions in depressive symptoms across baseline depression severity levels set the stage for sustainability and widespread clinical-community linkages. First, the clinical outcomes used in this study align with frequently used quality care metrics and performance improvement measures [e.g., National Center for Quality Assurance (NCQA) Healthcare Effectiveness Data and Information Set (HEDIS)], which incentivize routine screening and effective treatment (42–44). Second, the significant reductions in clinical remission and clinical response from PEARLS are comparable to or better than remission and response achieved with pharmacological care (45–48). This comparability is encouraging because pharmacological care may not be universally accessible to or preferred by many older adults because of the risk of side effects and adverse events (49) and resistance to anti-depressant treatment (50). Further, because PEARLS is easily accessible, it can mitigate social determinants of health that limit access to anti-depressants (e.g., food insecurity, housing) (51–53) or the stigma associated clinical depression care (54–57). PEARLS may offset these barriers to pharmacological care as a community-based treatment alternative that has comparable effectiveness and reaches older adults who have been underserved by clinical care. A public health approach (58, 59) is needed to raise awareness about depression given stigma from older adults, families, and providers; routinize depression screening to improve recognition and referral to quality care; and provide accessible, culturally and linguistically appropriate care that aligns with older adults’ preferences, resources, and values. This includes strengthening clinical-community partnerships for patient screening and program referral to address depression more holistically across community and clinical sectors; following recommendations from the Chronic Care Model given the high cost of managing multiple chronic conditions (of which depression is the leading cause of disability and interferes with managing other health issues) (59, 60).

In the current study, the number of PEARLS sessions attended was associated with significant reductions in depressive symptoms. Time to clinical remission showed a logical dose–response with PEARLS session attendance based on participants’ baseline depression levels. Intuitively, participants who entered the program with more severe depression at baseline required more PEARLS sessions to achieve clinical remission. However, the number of sessions needed to achieve clinical remission differed by the definition applied. On average, time to remission for those with severe depression was 4.65 sessions using the “no longer having one or both cardinal symptoms” definition and 7.12 sessions for the “reduced PHQ-9 scores to <5” definition. Conversely, on average, time to clinical response was consistent across baseline depression severity levels (range from 5.02 to 5.29 sessions), which suggests between five and six sessions are needed to reduce baseline PHQ-9 scores by ≥50%. This variability between outcome definitions reinforces the need for careful selection of clinically meaningful improvements by sites delivering PEARLS. Understanding the anticipated number of sessions needed to achieve clinically meaningful improvements can help sites maintain program fidelity (33) to retain PEARLS participants and ensure they receive an adequate intervention dose while reducing wait times for services (23, 27, 61).

### 4.1. Limitations

This multi-state applied study is not without limitations. First, PEARLS data were collected and analyzed from four U.S. states, which may not represent the intervention delivery infrastructure or participants reached in other states across the country. A primary strength of PEARLS is its potential to improve access to mental health services among underserved U.S. residents with depression; however, such service inequities may not be present in other Western countries because of universal healthcare. As such, despite the strength of these findings, they should be cautiously generalized to other populations. Future studies are recommended to examine the appropriateness and effectiveness of PEARLS in other U.S. communities and countries. Second, although previous randomized controlled trials have been conducted showing the effectiveness of PEARLS, this study only examined data from PEARLS participants; there were no comparison or control groups included in analyses. While this study demonstrated the effectiveness of PEARLS in “real world” practice within four distinct geographic areas, the single-group research design did not protect against all potential threats to external validity. Beyond the inclusion of a comparator group, researchers are encouraged to further investigate the program’s adaptability and accessibility in addressing late-life depression in diverse community settings. Third, there were missing data for gender, education level, income level, and marital status. Rather than omit cases, “unknown” categories were created for missing values for these covariates. While this allowed for the maximum number of cases to be retained in study analyses, this may have impacted the interpretation of findings. However, it should be acknowledged that only the “unknown” category for income level was significantly associated with depression symptom improvement in two Cox proportional hazard models in this study. Fourth, some contextual information and important variables were not available for this study. For example, while PEARLS delivery sites utilized a series of participant recruitment and engagement strategies, specific referral sources and marketing strategies used to attract participants were not analyzed. Further, beyond marital status, household composition and sources of social support were not provided or included in analyses. Researchers are encouraged to examine such contextual information and include these and other relevant variables in future studies.

## 5. Conclusion

Findings confirm that PEARLS can effectively improve depressive symptoms among older adults in “real world” community settings and assist large proportions of participants to achieve clinical remission and clinical response. Time to clinical response was consistent across baseline depression severity levels, showing the universal benefit of PEARLS to decrease depressive symptoms by 50% or more over time. While time to clinical remission was fastest among those with mild depression, participants with more severe depression may require more sessions or time in PEARLS to achieve remission. This study highlights the ability of PEARLS to meet different metrics and measures of success, which is important to delivery sites wanting to meet various clinical standards. Given its effectiveness, PEARLS is a strong intervention to provide community-based depression care to underserved older adults who may not be able to access or afford clinical care.

## Data availability statement

The data analyzed in this study is subject to the following licenses/restrictions: The datasets presented in this article are not readily available because they are based on a data use agreement and approval from funded sites where the data originated. Requests to access these datasets should be directed to MS, matthew.smith@tamu.edu.

## Ethics statement

The studies involving human participants were reviewed and approved by the Texas A&M University. Written informed consent for participation was not required for this study in accordance with the national legislation and the institutional requirements.

## Author contributions

MS and AM conceptualized the study and wrote the manuscript. LS wrote and critically reviewed the manuscript. CM obtained the study funding and critically reviewed the manuscript. MT facilitated data access and critically reviewed the manuscript. LZ critically reviewed the manuscript. AM performed formal statistical analyses. All authors contributed to the article and approved the submitted version.

## Funding

Funding for this study was provided by the Administration for Community Living to Florida Health Networks.

## Conflict of interest

The authors declare that the research was conducted in the absence of any commercial or financial relationships that could be construed as a potential conflict of interest.

## Publisher’s note

All claims expressed in this article are solely those of the authors and do not necessarily represent those of their affiliated organizations, or those of the publisher, the editors and the reviewers. Any product that may be evaluated in this article, or claim that may be made by its manufacturer, is not guaranteed or endorsed by the publisher.

## References

[1] Federal Interagency Forum on Aging-Related Statistics. Older Americans 2020: key indicators of well-being. Washington, DC: U.S. Government Printing Office (2020).

[2] Villarroel MA Terlizzi EP. Symptoms of depression among adults: United States, 2019. NCHS Data Brief. Hyattsville, MD: National Center for Health Statistics (2020) 379:1–8. 33054920

[3] Luppa M Sikorski C Luck T Ehreke L Konnopka A Wiese B. Age- and gender-specific prevalence of depression in latest-life – systematic review and meta-analysis. J Affect Disord. (2012) 136:212–21. doi: 10.1016/j.jad.2010.11.033, PMID: 21194754

[4] Zenebe Y Akele B Wselassie M Necho M. Prevalence and determinants of depression among old age: a systematic review and meta-analysis. Ann General Psychiatry. (2021) 20:55. doi: 10.1186/s12991-021-00375-x, PMID: 34922595 PMC8684627

[5] Schaakxs R Comijs HC van der Mast RC Schoevers RA Beekman ATF Penninx BWJH. Risk factors for depression: differential across age? Am J Geriatr Psychiatry. (2017) 25:966–77. doi: 10.1016/j.jagp.2017.04.004 28529007

[6] Ylli A Miszkurka M Phillips SP Guralnik J Deshpande N Zunzunegui MV. Clinically relevant depression in old age: an international study with populations from Canada, Latin America and Eastern Europe. Psychiatry Res. (2016) 241:236–41. doi: 10.1016/j.psychres.2016.04.096, PMID: 27183110

[7] Chapman DP Perry GS Strine TW. The vital link between chronic disease and depressive disorders. Prev Chronic Dis. (2005) 2:A14. Available at: http://www.cdc.gov/pcd/issues/2005/jan/04_0066.htm PMID: 15670467 PMC1323317

[8] Bor JS. Among the elderly, many mental illnesses go undiagnosed. Health Aff. (2015) 34:727–31. doi: 10.1377/hlthaff.2015.0314, PMID: 25941272

[9] Allan CE Valkanova V Ebmeier KP. Depression in older people is underdiagnosed. Practitioner. (2014) 258:19–22. PMID: 25065018

[10] Wei J Hou R Zhang X Xu H Xie L Chandrasekar EK. The association of late-life depression with all-cause and cardiovascular mortality among community-dwelling older adults: systematic review and meta-analysis. Br J Psychiatry. (2019) 215:449–55. doi: 10.1192/bjp.2019.74, PMID: 30968781

[11] Voros V Fekete S Tenyi T Rihmer Z Szili I Osvath P. Untreated depressive symptoms significantly worsen quality of life in old age and may lead to the misdiagnosis of dementia: a cross-sectional study. Ann General Psychiatry. (2020) 19:52. doi: 10.1186/s12991-020-00302-6, PMID: 32944058 PMC7493324

[12] Centers for Disease Control and Prevention. Depression is not a normal part of growing older. (2021). Available at: https://www.cdc.gov/aging/depression/index.html (Accessed February 15, 2023).

[13] Office of the Surgeon General, Center for Mental Health Services, and National Institute of Mental Health. Mental health: a report of the surgeon general. Rockville, MD: U.S. Department of Health and Human Services (1999).

[14] Williams SZ Chung GS Muennig PA. Undiagnosed depression: a community diagnosis. SSM Popul Health. (2017) 3:633–8. doi: 10.1016/j.ssmph.2017.07.012, PMID: 29349251 PMC5769115

[15] Karel MJ Gatz M Smyer MA. Aging and mental health in the decade ahead: what psychologists need to know. Am Psychol. (2012) 67:184–98. doi: 10.1037/a0025393, PMID: 21942364

[16] Ghio L Vaggi M Amore M Ferrannini L Natta W. Unmet needs and research challenges for late-life mood disorders. Aging Clin Exp Res. (2014) 26:101–14. doi: 10.1007/s40520-013-0149-z, PMID: 24078460

[17] Taylor WD. Should antidepressant medication be used in the elderly? Expert Rev Neurother. (2015) 15:961–3. doi: 10.1586/14737175.2015.1070671, PMID: 26196054 PMC4552589

[18] Allan CL Ebmeier KP. Review of treatment for late-life depression. Adv Psychiatr Treat. (2013) 19:302–9. doi: 10.1192/apt.bp.112.010835

[19] Snowden M Steinman L Frederick J. Treating depression in older adults: challenges to implementing the recommendations of an expert panel. Prev Chronic Dis. (2008) 5:A26. PMID: 18082015 PMC2248773

[20] Solway E Estes CL Goldberg S Berry J. Access barriers to mental health services for older adults from diverse populations: perspectives of leaders in mental health and aging. J Aging Soc Policy. (2010) 22:360–78. doi: 10.1080/08959420.2010.507650, PMID: 20924892

[21] Steinman LE Frederick JT Prohaska T Satariano WA Dornberg-Lee S Fisher R. Recommendations for treating depression in community-based older adults. Am J Prev Med. (2007) 33:175–81. doi: 10.1016/j.amepre.2007.04.034 17826575

[22] Program to Encourage Active. Rewarding lives (PEARLS). (2021). Available at: https://depts.washington.edu/hprc/evidence-based-programs/pearls/ (Accessed February 15, 2023).

[23] Ciechanowski P Wagner E Schmaling K Schwartz S Williams B Diehr P. Community-integrated home-based depression treatment in older adults: a randomized controlled trial. JAMA. (2004) 291:1569–77. doi: 10.1001/jama.291.13.1569, PMID: 15069044

[24] Steinman L Parrish A Mayotte C Bravo Acevedo P Torres E Markova M. Increasing social connectedness for underserved older adults living with depression: a pre-post evaluation of PEARLS. Am J Geriatr Psychiatry. (2020) 29:828–42. doi: 10.1016/j.jagp.2020.10.005, PMID: 33187883 PMC7564120

[25] Chaytor N Ciechanowski P Miller JW Fraser R Russo J Unutzer J. Long-term outcomes from the PEARLS randomized trial for the treatment of depression in patients with epilepsy. Epilepsy Behav. (2011) 20:545–9. doi: 10.1016/j.yebeh.2011.01.017, PMID: 21333607

[26] Ciechanowski P Chaytor N Miller J Fraser R Russo J Unutzer J. PEARLS depression treatment for individuals with epilepsy: a randomized controlled trial. Epilepsy Behav. (2010) 19:225–31. doi: 10.1016/j.yebeh.2010.06.003, PMID: 20609631

[27] Snowden MB Steinman LE Piering P Rigor S Yip A. Translating PEARLS: lessons learned from providers and participants. Front Public Health. (2014) 2:256. doi: 10.3389/fpubh.2014.00256, PMID: 25964932 PMC4410328

[28] Steinman LE Gasca A Hoeft TJ Raue PJ Henderson S Perez R. “We are the sun for our community:” partnering with community health workers/promotores to adapt, deliver and evaluate a home-based collaborative care model to improve equity in access to quality depression care for older U.S. Latino adults who are underserved. Front Public Health. (2023) 11:1079319. doi: 10.3389/fpubh.2023.1079319, PMID: 36817932 PMC9932325

[29] Steinman LE Parrish AT Kohn MJ Wu S Hara-Hubbard KK Brown L. Partnering with community-based organizations to improve equitable access to depression care for underserved older adults in the U.S.: qualitative formative research. Front Public Health. (2023) 10:1079082. doi: 10.3389/fpubh.2022.1079082 36793362 PMC9922751

[30] Quijano LM Stanley MA Petersen NJ Casado BL Steinberg EH Cully JA. Healthy IDEAS: a depression intervention delivered by community-based case managers serving older adults. J Appl Gerontol. (2007) 26:139–56. doi: 10.1177/0733464807299354

[31] Gitlin LN Harris LF Mc Coy M Chernett NL Jutkowitz E Pizzi LT. A community-integrated home based depression intervention for older African Americans: description of the beat the blues randomized trial and intervention costs. BMC Geriatr. (2012) 12:4. doi: 10.1186/1471-2318-12-4 22325065 PMC3293778

[32] PEARLS training and toolkit (2023). Available at: https://depts.washington.edu/hprc/programs-tools/pearls/get-started-with-pearls/ (Accessed February 15, 2023).

[33] Farren L Snowden M Steinman L Monroe-De VM. Development and evaluation of a fidelity instrument for PEARLS. Front Public Health. (2014) 2:200. doi: 10.3389/fpubh.2014.00200, PMID: 25964917 PMC4410416

[34] Kroenke K Spitzer RL Williams JBW. The PHQ-9: validity of a brief depression severity measure. J Gen Intern Med. (2001) 16:606–13. doi: 10.1046/j.1525-1497.2001.016009606.x, PMID: 11556941 PMC1495268

[35] American Psychiatric Association, DSM-5 Task Force. Diagnostic and statistical manual of mental disorders: DSM-5™ (5th ed.) Washington, DC: American Psychiatric Publishing, Inc. (2013) doi: 10.1176/appi.books.9780890425596

[36] Kroenke K Spitzer RL Williams J Löwe B. The patient health questionnaire somatic, anxiety, and depressive symptom scales: a systematic review. Gen Hosp Psychiatry. (2010) 32:345–59. doi: 10.1016/j.genhosppsych.2010.03.006, PMID: 20633738

[37] Laborde-Lahoz P El-Gabalawy R Kinley J Kirwin PD Sareen J Pietrzak RH. Subsyndromal depression among older adults in the USA: prevalence, comorbidity, and risk for new-onset psychiatric disorders in late life. Int J Geriatr Psychiatry. (2015) 30:677–85. doi: 10.1002/gps.4204, PMID: 25345806

[38] Smith ML Steinman LE Casey EA. Combatting social isolation among older adults in a time of physical distancing: the COVID-19 social connectivity paradox. Front Public Health. (2020) 8:403. doi: 10.3389/fpubh.2020.00403, PMID: 32850605 PMC7396644

[39] Chan YF Huang H Bradley K Unützer J. Referral for substance abuse treatment and depression improvement among patients with co-occurring disorders seeking behavioral health services in primary care. J Subst Abus Treat. (2014) 46:106–12. doi: 10.1016/j.jsat.2013.08.016, PMID: 24095002

[40] Vemer P Bouwmans C Zijlstra-Vlasveld MC van der Feltz-Cornelis CM Hakkaart-van RL. Let’s get back to work: survival analysis on the return-to-work after depression. Neuropsychiatr Dis Treat. (2013) 9:1637–45. doi: 10.2147/NDT.S49883, PMID: 24187499 PMC3810438

[41] Coley RY Boggs JM Beck A Hartzler AL Simon GE. Defining success in measurement-based care for depression: a comparison of common metrics. Psychiatr Serv. (2020) 71:312–8. doi: 10.1176/appi.ps.201900295, PMID: 31847739

[42] Amerigroup. HEDIS benchmarks and coding guidelines electronic clinical data systems (ECDS). (2022). Available at: https://provider.amerigroup.com/docs/gpp/TX_CAID_HEDISBenchmarksCodingECDS22.pdf?v=202207201839.

[43] National Committee for Quality Assurance. HEDIS depression measures specified for electronic clinical data systems. (2023). Available at: https://www.ncqa.org/hedis/the-future-of-hedis/hedis-depression-measures-specified-for-electronic-clinical-data/ (Accessed February 15, 2023).

[44] National Committee for Quality Assurance. Utilization of the PHQ-9 to monitor depression symptoms for adolescents and adults (DMS). (2023). Available at: https://www.ncqa.org/hedis/measures/utilization-of-the-phq-9-to-monitor-depression-symptoms-for-adolescents-and-adults/ (Accessed February 15, 2023).

[45] Gutsmiedl K Krause M Bighelli I Schneider-Thoma J Leucht S. How well do elderly patients with major depressive disorder respond to antidepressants: a systematic review and single-group meta-analysis. BMC Psychiatry. (2020) 20:102. doi: 10.1186/s12888-020-02514-2, PMID: 32131786 PMC7057600

[46] Mallery L MacLeod T Allen M McLean-Veysey P Rodney-Cail N Bezanson E. Systematic review and meta-analysis of second-generation antidepressants for the treatment of older adults with depression: questionable benefit and considerations for frailty. BMC Geriatr. (2019) 19:306. doi: 10.1186/s12877-019-1327-4, PMID: 31718566 PMC6852920

[47] Lindblad AJ Clarke J Lu S. Antidepressants in the elderly. Can Fam Physician. (2019) 65:340. PMID: 31088873 PMC6516699

[48] Nelson JC Delucchi K Schneider LS. Efficacy of second generation antidepressants in late-life depression: a meta-analysis of the evidence. Am J Geriatr Psychiatry. (2008) 16:558–67. doi: 10.1097/JGP.0b013e3181693288, PMID: 18591576

[49] Behlke LM Lenze EJ Carney RM. The cardiovascular effects of newer antidepressants in older adults and those with or at high risk for cardiovascular diseases. CNS Drugs. (2020) 34:1133–47. doi: 10.1007/s40263-020-00763-z, PMID: 33064291 PMC7666056

[50] Givens JL Datto CJ Ruckdeschel K Knott K Zubritsky C Oslin DW. Older patients’ aversion to antidepressants: a qualitative study. J Gen Intern Med. (2006) 21:146–51. doi: 10.1111/j.1525-1497.2005.00296.x, PMID: 16336620 PMC1484662

[51] Wilder ME Kulie P Jensen C Levett P Blanchard J Dominguez LW. The impact of social determinants of health on medication adherence: a systematic review and meta-analysis. J Gen Intern Med. (2021) 36:1359–70. doi: 10.1007/s11606-020-06447-0, PMID: 33515188 PMC8131473

[52] Cheruvu VK Chiyaka ET. Prevalence of depressive symptoms among older adults who reported medical cost as a barrier to seeking health care: findings from a nationally representative sample. BMC Geriatr. (2019) 19:192. doi: 10.1186/s12877-019-1203-2, PMID: 31319807 PMC6639933

[53] Gellad WF Grenard JL Marcum ZA. A systematic review of barriers to medication adherence in the elderly: looking beyond cost and regimen complexity. Am J Geriatr Pharmacother. (2011) 9:11–23. doi: 10.1016/j.amjopharm.2011.02.004, PMID: 21459305 PMC3084587

[54] Nair P Bhanu C Frost R Buszewicz M Walters KR. A systematic review of older adults’ attitudes towards depression and its treatment. Gerontologist. (2019) 60:e93–e104. doi: 10.1093/geront/gnz048 31115449

[55] Stark A Kaduszkiewicz H Stein J Maier W Heser K Weyerer S. A qualitative study on older primary care patients' perspectives on depression and its treatments - potential barriers to and opportunities for managing depression. BMC Fam Pract. (2018) 19:2. doi: 10.1186/s12875-017-0684-3, PMID: 29295706 PMC5751798

[56] Cruz M Pincus HA Harman J Reynolds CF Post EP. Barriers to care-seeking for depressed African Americans. Int J Psychiatry Med. (2008) 38:71–80. doi: 10.2190/PM.38.1.g, PMID: 18624019

[57] Sirey JA Bruce ML Alexopoulos GS Perlick DA Friedman SJ Meyers BS. Stigma as a barrier to recovery: perceived stigma and patient-rated severity of illness as predictors of antidepressant drug adherence. Psychiatr Serv. (2001) 52:1615–20. doi: 10.1176/appi.ps.52.12.1615, PMID: 11726752

[58] World Health Organization. Mental health of older adults. (2017). Available at: https://www.who.int/news-room/fact-sheets/detail/mental-health-of-older-adults (Accessed February 15, 2023).

[59] Centers for Disease Control and Prevention. CDC promotes public health approach to address depression among older adults. Available at: https://www.cdc.gov/aging/pdf/cib_mental_health.pdf (Accessed February 15, 2023).

[60] Krist AH O'Loughlin K Woolf SH Woolf SH Sabo RT Sabo RT. Enhanced care planning and clinical-community linkages versus usual care to address basic needs of patients with multiple chronic conditions: a clinician-level randomized controlled trial. Trials. (2020) 21:517. doi: 10.1186/s13063-020-04463-3, PMID: 32527322 PMC7291479

[61] Steinman L Hammerback K Snowden M. It could be a pearl to you: exploring recruitment and retention of the program to encourage active, rewarding lives (PEARLS) with hard-to-reach populations. Gerontologist. (2015) 55:667–76. doi: 10.1093/geront/gnt137, PMID: 24270214

